# Diagnostic delays in schizophrenia with catatonic symptoms mimicking conversive disorder

**DOI:** 10.1192/j.eurpsy.2025.1849

**Published:** 2025-08-26

**Authors:** J. L. P. Fernandes, N. Monteiro, S. Morais, J. F. A. Brás

**Affiliations:** 1 Centro de Responsabilidade Integrado de Psiquiatria, Unidade Local de Saúde de Coimbra; 2 Faculdade de Medicina, Universidade de Coimbra, Coimbra, Portugal

## Abstract

**Introduction:**

Notwithstanding the drastic reduction in the prevalence of catatonic symptoms in schizophrenia with the development of anti-psychotic treatment regimens since the 1950’s, a subgroup of patients still presents mostly such symptoms, associated with worse long-term prognosis.

**Objectives:**

Discuss the challenges surrounding the diagnosis and clinical management of patients with schizophrenia with catatonic symptoms.

**Methods:**

In addition to describing a case report of a male with catatonic symptoms mimicking conversive disorder, research was undertaken in PubMed and other databases using the keywords “conversive disorder, “catatonia” and “schizophrenia”.

**Results:**

A 29-year-old male patient, a former Geology BA student followed by Psychiatry in our hospital for conversive symptoms – namely mutism and sudden episodes of motor paralysis with no changes in the neurological examination or imaging exams - and admitted twice for that reason in the prior 6 months, was brought to the ED by police after an attempted self-injury with a knife. The family reported for the past week sleep-wake inversion, social isolation, compulsive smoking, refusal to eat any homemade food, soliloquies, staring at the walls for no reason and, in the last 3 days, post its with messages such as “I sinned” or “I disappointed God”. In the ER, the patient engaged in mutism, with negativism in his posture. Considering the high suicide risk and the presence of psychotic symptoms, including persecutory, poisoning and mystical delusions as well as likely auditive-verbal hallucinatory activity, he was admitted again, this time in involuntary regime. To exclude secondary causes, both blood samples and imaging techniques (first head CT and later MRI) were ordered. The bloodwork revealed increased levels of CK without any other significant findings, and imaging techniques also had negative results. In the psychiatric ward, the patient engaged in selective mutism towards the medical team, likely in a context of persecutory delusions. Due to the prominence of negative symptoms and lack of adhesion to treatment, we choose to treat with intramuscular paliperidone and sertraline, 50 mg. With this treatment, the patient started feeding himself again, resumed a daily routine and ceased his mutism. Yet, he remained highly defensive psychopathologically, with very poor speech content and severe affective blunting. He was discharged with the diagnosis of schizophrenia and has been followed in outpatient visitations, remaining clinically stable.

**Conclusions:**

Catatonic schizophrenia in its first presentation can be confused with conversive disorder, given the fact both may share movement disorders, in psychotic patients who collaborate very little with psychiatrists, requiring a careful combination of anamnesis, mental state examination and gathering of information with family members or others close to the patient.

**Disclosure of Interest:**

None Declared

